# Epigenetics in Personality Disorders: Today's Insights

**DOI:** 10.3389/fpsyt.2018.00579

**Published:** 2018-11-19

**Authors:** Dorothee Maria Gescher, Kai G. Kahl, Thomas Hillemacher, Helge Frieling, Jens Kuhn, Thomas Frodl

**Affiliations:** ^1^Department of Psychiatry and Psychotherapy, Otto-von-Guericke University of Magdeburg, Magdeburg, Germany; ^2^Department of Psychiatry, Social Psychiatry and Psychotherapy, Hanover Medical School, Hanover, Germany; ^3^Department of Psychiatry, Paracelsus Medical University, Nuremberg, Germany; ^4^Department of Psychiatry and Psychotherapy, University of Cologne, Cologne, Germany; ^5^German Centre for Neurodegenerative Diseases, Magdeburg, Germany

**Keywords:** epigenetic aberrations, personality disorder, personality trait, early childhood adversity, aggression, antisocial, molecular pathobiology, personalized therapy

## Abstract

**Objective:** Epigenetic mechanisms have been described in several mental disorders, such as mood disorders, anxiety disorders and schizophrenia. However, less is known about the influence of epigenetic mechanisms with regard to personality disorders (PD). Therefore, we conducted a literature review on existing original data with regards to epigenetic peculiarities in connection with personality disorders.

**Methods:** Systematic literature review using PRISMA guidelines. Search was performed via NCBI PubMed by keywords and their combinations. Used search terms included “epigenetic,” “methylation,” “acetylation” plus designations of specified personality traits and disorders according to DSM-IV.

**Results:** Search yielded in total 345 publications, 257 thereof with psychiatric topic, 72 on personality disorder or traits, 43 of which were in humans and epigenetic, 23 thereof were original studies. Lastly, 23 original publications fulfilled the intended search criteria and were included. Those are 13 studies on gene methylation pattern with aggressive, antisocial and impulsive traits, 9 with borderline personality disorder (BPD), and 2 with antisocial personality disorder (ASPD). The results of these studies showed significant associations of PD with methylation aberrances in system-wide genes and suggest evidence for epigenetic processes in the development of personality traits and personality disorders. Environmental factors, of which childhood trauma showed a high impact, interfered with many neurofunctional genes. Methylation alterations in ASPD and BPD repeatedly affected *HTR2A, HTR3A, NR3C1*, and *MAOA* genes.

**Summary:** Epigenetic studies in PD seem to be a useful approach to elucidate the interaction of co-working risk factors in the pathogenesis of personality traits and disorders. However, the complexity of pathogenesis leads to divergent results and impedes an explicit interpretation. Differing methylation patterns within the selected PD could indicate subgroups which would benefit from patient-oriented therapeutic adjustments. They might play a major role in the future design and observation of early therapeutic intervention and thus could help to prevent severe dysfunctional conduct or full-blown personality disorder in risk subjects.

## Introduction

The epigenetic view on genes presumably associated with psychiatric disorders is gaining increasing academic interest and enables auxiliary insights in the pathogenesis of a particular disease. Severe psychiatric axis-I disorders like major depressive disorder (MDD) are currently widely investigated at the epigenetic level, and results account for a substantial pathogenetic impact of gene epigenetic modifications.

Epigenetic mechanisms in general function to homeostatically control gene accessibility and transcriptional functionality. Thus, gene function can be organized in both a highly programmed and life-enduring, but also in an environmentally-responsive way (1). The transcriptome as the immediate representation of genomic activity is regulated by (i) control of gene access for the transcriptional machinery through chromatin condensation and histone modification such as (de-)acetylation, (de-)phosphorylation, sumoylation, (ii) non-coding and microRNA that influences chromatin formation, as well as RNA translation and degradation, and (iii) covalent DNA changes by methylation of the cytosine nucleotide, which hampers transcriptase accessibility to the methylated region, and which can activate enzymes that interact in silencing the specific gene [reviewed in (2)].

Whereas epigenetic patterns stably determine mitosis-persistent cell differentiation as a precondition for embryonal development without changing DNA sequence, the epigenome also represents a dynamic adaption to environmental conditions. There is growing evidence in the last two decades that early life experience can affect long standing somatic and mental health trajectories in animals and humans by influencing the epigenetic pattern and thus affects structure and accessibility of the genome. Studies in rodents demonstrated the decisive impact of maternal care and early social adversity on the offspring's development and adult phenotypes. Early life adversity (ELA) in rats led to increased hippocampal glucocorticoid receptor (GR) expression, disturbed hypothalamus-pituitary-adrenal (HPA) axis functionality, and changed DNA methylation of the GR gene (*NR3C1)* in the hippocampus (3), of the brain-derived neurotropic factor gene (*BDNF*) promoter in the prefrontal cortex (4) and of the Arginine Vasopressin gene (*AVP*) (5). In humans, ELA was associated with decreased GR mRNA expression and *NR3C1* hypermethylation in post-mortem human brains (6). Hitherto findings indicate that epigenetic patterns found in context with ELA in animals and humans are not restricted to suggestive disease-associated functional genes but are spread genome-wide (7, 8), and are not stringently tissue-specific (9), since peripheral blood cells (PBC), especially T-cell lymphocytes, were shown to reflect epigenetic patterns similar to neuronal cells in culture and in brain tissue (9–11).

The translation of environmental signals into epigenetic information can be triggered by neuronal activity that initiates intracellular pathways such as cAMP signaling mediated histone acetylation or that influences and interacts with other epigenetic processes (12–14). Additionally, activity of the *AVP* promoter is regulated by the methyl-CpG-binding protein 2 (MeCp2), which is phosphorylated and activated by depolarization of hypothalamic neurons and in turn moderates demethylation of the *BDNF* promoter (5, 15, 16). In sum, although many details of molecular mechanisms remain unknown, the present insights substantiate and refine the idea how environmental signals might be translated into intracellular information and molecular memory.

Currently, a considerable number of studies explore epigenetic changes in association with behavior or affect difficulties like aggression or fear in human and animal subjects, particularly in connection with disturbances of the serotonergic system that meanwhile is well-known to be crucial in early brain development. The objective of this work was to review the current original publications on epigenetic modifications associated with personality disorders (PD) in humans.

## Methods

Literature search was performed as a systematic review according the Preferred Recording Items for Systematic Reviews and Meta-Analyses (PRISMA-P) guidelines (17). We based our search on the PubMed Central database of the U.S. National Institutes of Health's National Library of Medicine (NIH/NLM) using terms oriented on the Medical Subject Headings (MeSH) of the NCBI Library.

For the search keywords were inserted in a double or triple combination to yield comprehensive hits. The following keywords were utilized: “personality,” “personality disorder,” “personality trait,” each of them combined with “epigenetic,” “methylation,” “acetylation,” “phosphorylation,” “ubiquitation,” “sumoylation,” “microRNA,” “chromatin” and “chromatin remodeling,” respectively, as well as with one of the keywords “aggression,” “anankastic,” “antisocial,” “anxious,” “avoidant,” “borderline,” “dependent,” “eccentric,” “emotionally unstable,” “histrionic,” “passive-aggressive,” “impulsive,” “narcissism,” “narcissistic,” “paranoid,” “schizoid,” and “schizotypal,” respectively. The search included publications until May 15th 2018.

**Figure 1:**
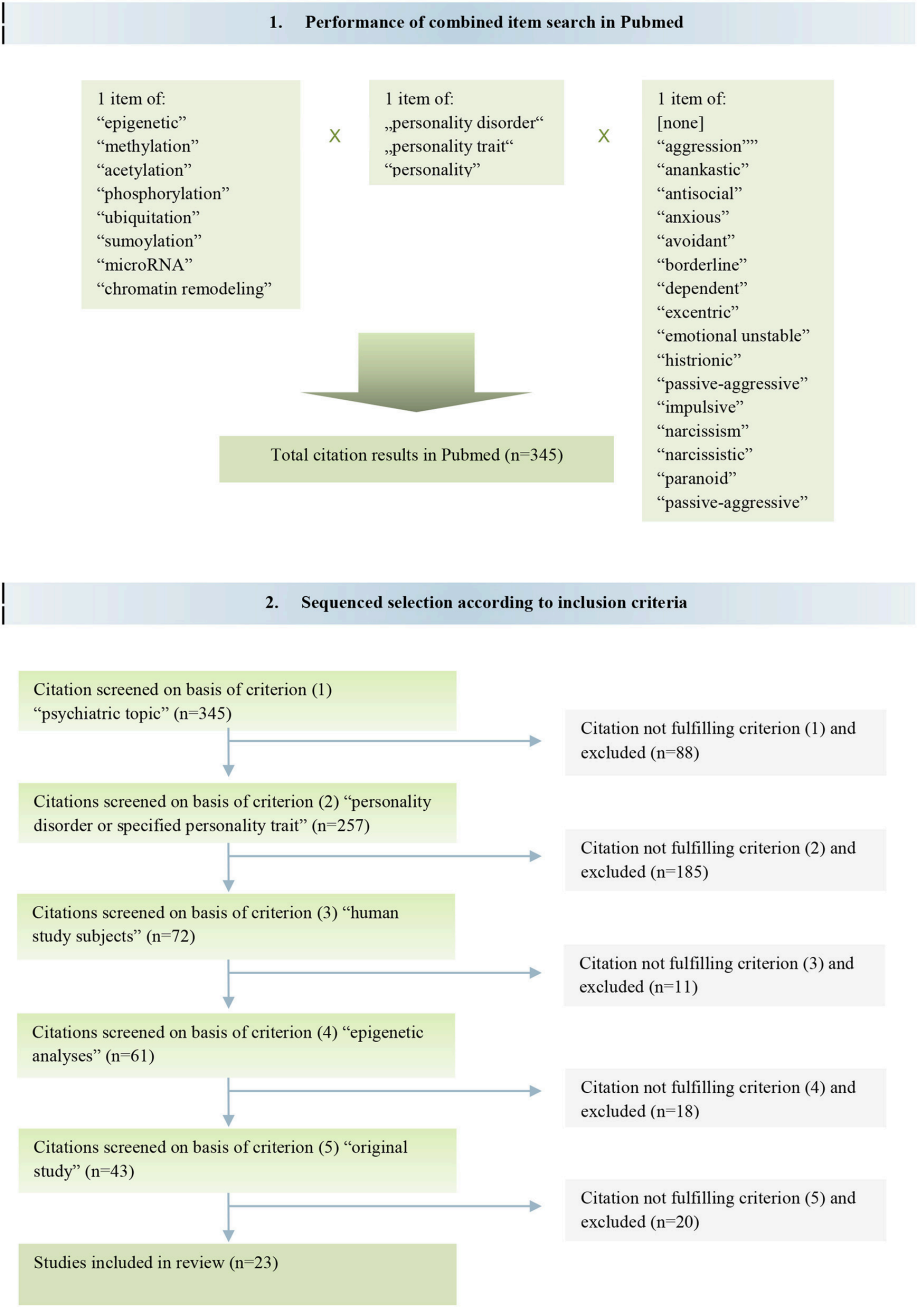
Flow diagram of study search and sequenced selection.

In total, the search yielded 345 different articles. We secondly perused the gained articles by reviewing their titles, abstracts and full texts in order to identify the proper articles matching to our literal research question. Therefore, studies were sequentially selected if they met the criteria (1) psychiatric topic [*n* = 257], (2) personality disorder or specified personality trait [*n* = 72 of (1)], (3) human study subjects [*n* = 61 of (2)], (4) epigenetic analyses [*n* = 43 of (3)], and (5) original study [*n* = 23 of (4)]. Following these selection criteria, it remained 23 articles according to the intended objective of this review (Figure 1).

Concomitantly, we used the PubMed search function of f1000prime (Faculty of 1000 Limited, London, UK). Herewith we found one further expedient study that met all of the described inclusion criteria (18). Finally, 24 original studies were included in this review.

## Results

Among the included studies, 13 explored the epigenetic influence on PT (two on impulsiveness, six on antisocial traits, seven on aggression) and 11 studied epigenetic differences in personality disorders (two in antisocial personality disorder (ASPD), 9 in borderline personality disorder (BPD)).

Size, constitution of study groups, and the number of investigated genes varied between the studies and ranged from single gene assays to genome-wide methylation analyses (GWA) with implications for the statistical power.

### Personality disorders

#### Antisocial personality disorder

With regards on antisocial PD, only methylation of the monoamine oxidase A gene (*MAOA*) has been examined. The two studies differed decisively with respect to study design and methylation results.

Philibert et al. considered the known variable nucleotide repeat (VTNR) region of *MAOA* and introduced a new VTNR region upstream of the transcriptional start site (TSS) of the gene (*MAOA* VTNR P2). They found a genotype-dependent methylation level and gene activity, but only in females (19). Within a total of well characterized 571 subjects (312 female) of the Iowa Adoption Study (IAS) they measured ASPD lifetime symptom counts in a linear mode according to DSM-IV criteria. Methylation patterns were analyzed at two promoter-associated CpG islands of *MAOA* in DNA extracted from EBV transformed lymphoblast cell lines from peripheral blood. Sequence analyses of the VTNR P2 revealed five genotypes with each seven to 11 eleven repeats (7R, 8R, 9R, 10R, 11R), of which the 9R genotype showed the lowest methylation in homozygous females, and the greatest gene activity in the functional analysis via luciferase essay. Presence of the low activity allele 10R was associated with higher vulnerability to the harming effects of childhood sexual and physical abuse and it accounted significantly for variances in symptom severity of ASPD in women. In male subjects no significant effect of the P2 genotype on *MAOA* methylation status was found.

In contrast, in a population of incarcerated men (*n* = 86) fulfilling the DSM-IV criteria for ASPD, Checknita et al. found a significant overall hypermethylation of the *MAOA* promoter region in the ASPD group compared with healthy controls (*n* = 93) with significant differences in methylation levels at 34 of 71 distinct *MAOA* promoter CpG sites. In their analysis, they did not consider symptom severity or childhood adversity. Methylation of *MAOA* promoter was associated with decreased gene activity (luciferase-assay) and positively correlated with 5-hydroxytryptophane levels in blood, thus suggesting functional relevance (20).

#### Borderline personality disorder

With respect to epigenetic modifications in BPD, different genes were suggested to be involved during individual development as well as in phenotypic characteristics of the disorder (Table 1). Apart from genome wide analyses (GWA), the main focus of theory-driven epigenetic studies was in gene regions coding for BDNF, glucocorticoid receptor (*NR3C1*), dopamine and serotonin receptors, MAOA, and catechol-O-methyltransferase (*COMT*). Most of the studies that focused on targeted genes considered a history of an early child trauma as a confounding factor in their analyses. The diversity in control group definition in the individual studies reflects the struggle for a suitable study design that allows for isolating disorder-specific characteristics. Methylation aberrations were mostly evaluated with diagnostic, but also with predictive concerns (21).

**Table 1:** Studies on epigenetics in personality disorders.

**Author**	**Year**	**PD**	**Gene**	**Co-factors**	**Results**	**Sample**	**Tissue/DNA**	**Limitations**
Philibert et al. (19)	2011	ASPD	*MAOA*	VNTR-genotype, CA	Identification of five genotypes of *MAOA* VNTR P2 (7R, 8R, 9R, 10R, 11R), 9R allele with the lowest methylation and associated with the highest activity (luciferase assay), 10R allele the lowest. Methylation status of *MAOA* promoter was genotype-dependent. VNTR genotype interacted with CA in predicting ASPD features. The presence of the low activity allele 10R at P2 was associated with significantly greater vulnerability to the impact of CA regarding ASPD symptoms. All results were found in women only.	*Subjects:* adult participants of the Iowa Adoptee Study (IAS), (*n* = 571, 312 female). *Diagnostics:* DSM-IV, CMI (SA, PA), ASPD lifetime symptom count (0–7). *Methods:* DNA bisulfite treatment, PCR amplification and methylation quantification. Selected methylation analysis of *MAOA* at two promoter-associated CpG islands (18 and 70 CpG sites, respectively). Sequencing VTNR genotype.	EBV-transformed lymphoblast cell lines
Checknita et al. (20)	2015	ASPD	*MAOA*		Methylation level of *MAOA* promoter region was significantly higher in ASPD subjects. Methylation in 34 of 71 CpG sites was significantly associated with ASPD, 31 of which were hypermethylated. *MAOA* promoter methylation was *in vitro* associated with decreased gene activity (luciferase-assay) and was positively associated with 5-HT plasma levels *in vivo*.	*Subjects:* incarcerated men (*n* = 86) with ASPD, HC (*n* = 93). *Diagnostics:* DSM-IV, SCID-I/-II. *Methods:* Genomic DNA bisulfite treatment. Selected methylation analysis of *MAOA* promoter (71 CpG sites, region chrX:4351407-43515991) performed by quantitative mass spectroscopy. *In vitro* functional analysis (luciferase assay). HPLC-analysis of 5-HT serum levels in blood in a subsample (*n* = 80).	PBC	No consideration of ELA.
Perroud et al. (21)	2013	BPD	*BDNF*	CT	*BDNF* methylation was significantly higher in both exon 1 and exon 4 CpG regions in BPD than in control subjects. The number of CT was significantly positive correlated with methylation levels. In BPD, *BDNF* methylation was significantly increased after I-DBT in both CpG-regions. This increase was decisively caused by the I-DBT nonresponders. I-DBT responders showed a decrease of methylation. No significant association of *BDNF* methylation with protein plasma levels.	*Subjects:* outpatients with BPD (*n* = 115, 108 female), HC (*n* = 52, 24 female). All patients received psychotropic medication. *Diagnostics:* DSM-IV, SCID-II, BDI-II, BHS, BIS-10, CTQ. *Methods:* intervention with I-DBT for 4 weeks. Methylation analysis of two CpG regions within *BDNF* exon 1 (9 CpG; chr11:27743473–27744564) and exon 4 (17 CpG; chr11:27723060–27723294). DNA bisulfite treatment, PCR amplification, methylation analysis by high-resolution melt assay. Quantizing BDNF plasma protein levels (ELISA).	Peipheral blood leukocytes	BPD subjects were on psychotropic medication. In HC, no association analysis of CT with methylation status.
Martín-Blanco et al. (22)	2014	BPD	*NR3C1*	CT	Overall methylation of *NR3C1* was significantly positively correlated with CT (in particular PA) and with clinical severity of BPD (in particular DIB-R total score and previous hospitalizations). No significant association between *NR3C1* methylation level and general severity of CT. Methylation of individual CpG sites showed single significance for association with PA and EN (CpG1-3) and with EA (CpG6).	*Subjects:* female outpatients with BPD (*n* = 281) without current episode of any axis I disorder. *Diagnostics:* DSM-IV, SCID-II, DIP-R, CTQ-SF. *Methods:* DNA bisulfite treatment, PCR amplification and pyrosequencing for selected methylation analysis of *NR3C1* exon 1F (8 CpG sites).	Peipheral blood leukocytes	Only female subjects. No control.
Perroud et al. (23)	2011	BPD	*NR3C1*	CT	Subjects with BPD, even without CT, showed higher *NR3C1* methylation levels than MDD subjects with the same reported CT. In BPD, childhood SA, PN, and number of types of CT were significantly associated with increased *NR3C1* methylation. No significant association was found between methylation status and childhood PA, EA and EN. In the whole sample, SA, PA, PN, EA, and EN were significantly associated with *NR3C1* methylation. Number of types of CT was significant positively correlated with overall methylation level.	*Subjects:* patients with BPD (*n* = 101, 95 female), MDD (*n* = 99, with low rate of abuses and neglect) and MDD with comorbid PTSD (*n* = 15). All subjects received psychotropic medication. *Diagnostics:* DSM-IV, SCID-II, DGIS, CTQ, BDI-II, clinical interview for detailed characteristics of suffered abuse. *Methods:* DNA bisulfite treatment, PCR amplification and pyrosequencing for selected methylation analysis of *NR3C1* exon 1F (8 CpG sites). Sample validation by confirmation analyses.	Peipheral blood leukocytes	All participants received psychotropic medication. No HC.
Groleau et al. (24)	2014	BPD	*DRD2*	CA	*Impact of BPD:* Significant group effect of methylation levels with BPD status. Mean methylation levels of *DRD2* in BSD/BPD subjects were significantly increased compared to NE, and marginally significant increased compared to BSD/non-BPD. No significant differences between total BSD and NE. Slightly significant (by trend) higher methylation of BSD/SA than NE (but not of BSD/PA).	*Subjects:* female outpatients with BSD (*n* = 52, 8 of which with BPD) and NE (*n* = 19). BSD subjects: *n* = 33 (63.5%) BN-purging subtype, *n* = 2 (3.8%) BN-non purging subtype, *n* = 17 (32.7%) ED n.o.s. *Diagnostics:* DSM-IV, EDE, CTI, SCID-II. *Methods:* DNA bisulfite treatment, PCR amplification and pyrosequencing for selected methylation analysis of *DRD2* exon 1 promoter (10 CpG sites; chr11:113346237-113346328).	PBC	Only female subjects. No HC.
Perroud et al. (25)	2016	BPD	*HTR3A*	SNP genotype, CT	*Severity of disease:* in BPD, BN and ADHD, CT was associated with higher severity of the disease across and within each disorder. BPD subjects showed highest CTQ scores. Particular methylation differences between disorders: in BPD subjects, methylation of specific CpG-sites was significantly increased. In ADHD subjects, two different CpG-sites were significantly hypermethylated (and significantly less methylated in BPD). *Impact of HTR3A genotype on methylation status:* CC carriers showed significantly higher methylation at a specific CpG-site than T allele carriers, independent of CT history. *Impact of CT on methylation status in BPD:* CTQ total and PA were significantly associated with *HTR3A* methylation at different individual CpG-sites. *Impact of methylation on BPD severity:* methylation at cpG2_III was significantly associated with nearly all criteria of BPD severity, CpG5_III with age of BPD onset and number of mood episodes, methylation at CpG1_III was significantly associated with psychotic symptoms.	*Subjects:* outpatients with BPD (*n* = 116, 106 female), BN (*n* = 122, 65 female), ADHD (*n* = 111, 33 female). All subjects were on stable psychopharmacological treatment. *Diagnostics:* DSM-IV, CTQ, SCID-II (BPD), WURS (ADHD), ASRS. *Methods:* DNA bisulfite treatment, PCR amplification and pyrosequencing for selected methylation analysis of *HTR3A* (8 CpG sites). Gene sequencing of rs1062613 variants.	PBC	All participants received psychotropic medication. No HC.
Dammann et al. (26)	2011	BPD	*COMT, HTR2A, MAOA, MAOB, NR3C1*		Average and individual CpG-site methylation levels of each *HTR2A, NR3C1, MAOA*, and *COMT* were significantly increased in BPD subjects, *MAOB* by trend. Significantly higher methylation was found in most of the analyzed single CpG sites: *HTR2A* (4 CpG sites), *NR3C1* (2 of 8 CpG sites), *MAOA* (1 of 5 CpG sites), *MAOB* (1 of 6 CpG sites) and *COMT* (2 of 4 CpG sites).	*Subjects:* outpatients with BPD (*n* = 26, 24 female) and HC (*n* = 11, all female). *Diagnostics:* DSM-IV. *Methods:* DNA bisulfite treatment, PCR amplification for selective methylation analysis: bisulfite pyrosequencing of *HTR2A, NR3C1, MAOA, MAOB* and *COMT* promoter regions (27CpG sites), performed in triplicates and averaged.	PBC	Nearly all subjects female. No consideration of ELA.
Teschler et al. (27)	2013	BPD	genome-wide		Methylation differences between BPD and HC subjects comprised a total of 256 significantly hypermethylated CpG sites in BPD, but significance of any of each didn't persist post Bonferroni correction. By selected quantitative analysis, hypermethylation in BPD was significant at particular CpG sites respecting *APBA2, APBA3, KCNQ1, MCF2, NINJ2, GATA4* compared with HC subjects (*GATA4* was significantly increased only in the bead chip assay).	*Subjects:* outpatients with BPD (*n* = 24, all female) and HC (*n* = 11). *Diagnostics:* DSM-IV, SCID-II. *Methods:* DNA bisulfite treatment, GWA by BeadChip technology, quantitative pyrosequencing.	PBC	Only female subjects. No consideration of ELA.
Teschler et al. (28)	2016	BPD	*PRIMA1 rDNA*		Methylation of the *rDNA* and *PRIMA1* promoter were significantly associated with BPD. In *PRIMA1* all of 6 CpG sites were each significantly hypermethylated in BPD subjects. All of 9 CpG sites within the *rDNA* promoter were each significantly less methylated in BPD subjects.	*Subjects:* outpatients with BPD (*n* = 24, all female) and HC (*n* = 11). *Diagnostics:* DSM-IV, SCID-II. *Methods:* DNA bisulfite treatment, pyrosequencing for selective methylation analysis of *rDNA* 5′ETS (5 CpG sites) and promoter (9 CpG sites), and of *PRIMA1* promoter CpG island (6 CpG sites).	PBC	Only female subjects. No consideration of ELA. All BPD subjects received psychotropic medication.
Prados et al. (29)	2015	BPD	genome-wide	CT	*Methylation differences between MDD and BPD subjects:* most significant results respected CpG sites associated with *EFNB1, SPSB2* and *CST9L* (multivariate analysis), and with *miR124-3, WDR60* and *FAM163A* all with *decreased* methylation in BPD subjects (univariate analysis). A high number of aberrantly methylated CpG sits were localized on chromosome X with *higher* methylation and on chromosome 6 with *lower* methylation in BPD. *Impact of CT:* CpG sites significantly associated with CTQ total score were located p.e. in *OCA2, MFAP2, CST9L, EP400, KCNQ2, A2ML, NT5DC2* and *RPH3L* (multivariate analysis) and within IL17RA exon 1, chromosome 6p22.1, and miR137 (highest significance) (univariate analysis). Methylation of *miR124-3* was associated with both severity of CA (higher methylation) and with BPD (lower methylation).	*Subjects:* outpatients with BPD (*n* = 96, 88 female) or with MDD (*n* = 93, 59 female). MDD subjects were selected for low rate of child abuse and neglect by CTQ. *Diagnostics:* DIGS, SKID-II, CTQ. CTQ, history of maltreatment was validated by a defined clinical talk, BIS-10, STAXI, TCI. *Methods:* DNA (*n* = 29 BPD, *n* = 18 MDD); GWA (485 577 CpGs). Specific methylation analysis of cg04927004 (chr20: 61′809′348) (near miR124-3): bisulfite-pyrosequencing performed in duplicates.	Peripheral blood leukocytes	No HC.

*5′ETS, 5′ external transcribed spacer; 5-HT, 5-hydroxytryptophane; ADHD, attention deficit hyperactivity disorder; A2ML, alpha-2 macroglobulin-like protein; APBA2(3), Amyloid Beta Precursor Protein Binding Family A Member 2(3) gene; ASPD, Antisocial personality disorder; ASRS, Adult ADHD Self-Report Scale; BDI, Beck Depression Inventory; BDNF, brain-derived neurotrophic factor; BHS, Beck Hopelessness Scale; BIS-10, Barrat Impulsivity Scale; BN, bulimia nervosa; BPD, borderline personality disorder; BSD, bulimia spectrum disorder; CA, childhood abuse; CMI, Childhood Maltreatment Index; COBRA, combined bisulfite restriction analysis; s-COMT, soluble catechol-O-methyltransferase gene; COBRA, Combined Bisulfite Restriction Analysis; CST9L, cystatin 9-like gene; CT, childhood trauma; CTI, Childhood Trauma Interview; CTQ(-SF), Childhood Trauma Questionnaire (Short Form); DIGS, Diagnostic Interview for Genetic Studies; DIP-R, Revised Diagnostic Interview for Borderlines; DRD2, dopamine receptor D2 gene; EA, emotional abuse; EDE, Eating Disorder Examination Interview; EFNB1, Ephrin B1 gene; ELA, early life adversity; ELISA, enzyme-linked immunosorbent assay**;** EN, emotional neglect; EP400, E1A binding protein P400 gene; FAM163A, family with sequence similarity 163 member A gene; GATA4, GATA binding protein 4 gene; GWA, genome-wide methylation analysis; HC, healthy control; HPLC, high performance liquid chromatography; HTR2A, serotonin receptor 2A gene; HTR3A, serotonin receptor 3A gene; I-DBT, Intensive Dialectical Behavioural Therapy; IAS, Iowa Adoptee Study; KCNQ1, potassium voltage-gated channel subfamily Q member 1 gene; MAOA(-B), monoamine oxidase A(B) gene; MDD, major depressive disorder; MFAP2, microfibrillar associated protein 2 gene; miR137, microRNA 137 gene; miR124-3, microRNA 124-3 gene; NE, no eating disorder; NINJ2, ninjurin 2 gene; NR3C1, nuclear receptor subfamily 3 group C member 1 gene (glucocorticoid receptor gene); NT5DC2, 5'-nucleotidase domain containing 2 gene; OCA2, oculocutaneous albinism II gene; PA, physical abuse; PBC, peripheral whole blood cells; PCR, polymerase chain reaction; PN, physical neglect; PRIMA1, proline rich membrane anchor 1 gene; PTSD, posttraumatic stress disorder; RPH3L, rabphilin 3A-like gene; SA, sexual abuse; SCID-I/-II, Structured Clinical Interview I/II for DSM-IV Personality Disorders; SPSB2, splA/ryanodine receptor domain and SOCS box containing 2 gene; STAXI, State-Trait Anger Expression Inventory; TCI, Temperament and Character Inventory; VTNR, variable number tandem repeats; WDR60, WD repeat domain 60 gene; WURS, Wender Utah ADHD Rating Scale*.

Perroud et al. studied methylation status of the *BDNF* gene in peripheral blood leucocytes and its modulation by a focused therapeutic intervention with intensive dialectical behavior therapy (I-DBT) comprising 4 weeks in outpatients with BPD (*n* = 115). Contrasted with healthy subjects, CpG-rich regions in exon 1 and exon 4 were significantly more highly methylated in BPD subjects before the therapeutic intervention. The number of different types of childhood trauma (CT) according the Childhood Trauma Questionnaire (CTQ) correlated positively with the mean methylation percentage of both CpG-regions. After intensive DBT, methylation status of the considered CpG sites was significantly increased in BPD patients. This effect could be traced back to the non-responders, whereas the responders showed a methylation decrease. However, the comparison of *BDNF* methylation status with peripheral serum protein levels of BDNF revealed no significant association (21).

Regarding studies on *NR3C1* methylation and PD, two studies focused on exon 1F promoter, which is functionally crucial. In a cohort of BPD outpatients (*n* = 281) Martin-Blanco et al. (22) found a significant positive correlation between overall *NR3C1* exon 1F methylation level of peripheral blood leucocytes and clinical severity. Exon 1F methylation was further significantly associated with childhood physical abuse. Individual CpG sites were associated with particular subscores of CTQ.

Perroud et al. (23) considered that a current severe mental illness might have epigenetic implications *per se* and could confound analyses that aim to isolating methylation characteristics specific for BPD. Therefore, in a comparison of subjects with BPD (*n* = 101) to those with MDD (*n* = 99) their results showed higher overall *NR3C1* exon 1F methylation levels in BPD than in MDD subjects in peripheral blood leucocytes. Further, methylation was associated with CT scaled by the CTQ, and correlated with childhood sexual, physical and emotional abuse, and physical and emotional neglect, respectively, and the number of these types of CT. *NR3C1* exon 1F hypermethylation in subjects with BPD was still significant when corrected for childhood maltreatment.

Regarding monoamine receptor genes, within a large-scaled study on subjects with bulimia spectrum disorders (BSD) with and without comorbid BPD Groleau et al. (24) found significant but marginally increased methylation of the dopamine D2 receptor gene (*DRD2*) exon 1 promoter region in whole peripheral blood cell (PBC) DNA of subjects with BSD and BPD compared with that of subjects with BSD only.

Methylation of the serotonin receptor 3A gene (*5HTR3A*) was found by Perroud et al. (25) to be correlated to clinical severity of BPD and other psychiatric disorders. The authors compared PBC DNA methylation levels of eight CpG sites within the 5HT3A gene in subjects with BPD (*n* = 116), attention deficit hyperactivity disorder (ADHD) (*n* = 111) and bipolar disorder (BD) (*n* = 122). They also considered single nucleotide variants (SNP) of the gene and CT history as additional factors and explored associations with methylation levels of the particular CpG sites.

Methylation levels between the patient groups differed significantly in most of the CpG sites and showed a distinct pattern of hyper- and hypomethylation in the several disorders in selected CpG sites. For BPD, subjects showed the highest scores in the CTQ and the highest methylation level between the patient groups. CT was associated with mean methylation status, and CTQ total score and physical abuse each with different selected CpG sites. CT was further associated with higher severity of disease. Carrying the CC-allele was significantly associated with methylation at one specific CpG site independent of CT (in all disorders).

Dammann et al. (26) analyzed five neuropsychiatric genes assumed to be of significance for psychopathological phenotype, in particular genes coding for soluble catechol-O-methyltransferase (*s-COMT*), serotonin receptor 2A (*HTR2A*), *NR3C1*, and X-chromosomal *MAOA* and *MAOB*. Methylation levels of each gene promoter were quantified in PBC DNA of individuals with BPD (*n* = 26, 24 female) and in healthy controls (*n* = 11, all female). In comparison to healthy controls, quantitative DNA methylation analysis showed significant elevated overall methylation levels in BPD subjects within *HTR2A, NR3C1*, and *s-COMT*. Gene methylation of *MAOA* and *MAOB* could only be analyzed in female subjects, and methylation of *MAOA* was significantly higher in BPD (of *MAOB* only by trend). Considering all 27 individual CpG sites, across the five genes that were investigated, average methylation level across all quantified regions was significantly higher in BPD patients compared to controls. Implications of attendant data as trauma history of the subjects weren't presented as part of the study, but it was noted that aberrant methylation had not been associated with traumatic experience in appropriate statistical tests.

Meanwhile epigenetic assays include genome-wide association studies (GWA) that indicate specific CpG sites of aberrant methylation levels across the whole genomic DNA and facilitate comprehensive aspects within epigenetic evaluations.

In extension to the aforementioned study, Teschler et al. (27) performed a GWA in PBC DNA between female BPD (*n* = 24) and HC (*n* = 11) subjects. Results showed a total of 256 significantly hypermethylated CpG sites in BPD, but significance of any of each didn't persist post Bonferroni correction. The research group selected seven hypermethylated genes for validation analyses and could endorse increased methylation in five of the genes, with specified CpG sites related to the amyloid beta (A4) precursor protein-binding family A member 2 (*APBA2*) and member 3 (*APBA3*) genes, potassium voltage-gated channel KQT-like subfamily member 1 gene (*KCNQ1*), MCF2 cell line derived transforming sequence gene (*MCF2*) and the ninjurin 2 gene (*NINJ2*). GATA binding protein 4 (*GATA4*) and holocarboxylase synthetase (*HLCS*) genes showed increased methylation in BPD in the GWA, but not in the validation analysis.

Methylation studies of the ribosomal RNA gene (*rDNA*) promoter, 5′ external transcribed spacer gene (*5*′*ETS*) and of the proline rich membrane anchor 1 gene (*PRIMA1*) promoter in PBC DNA of female subjects with BPD (*n* = 24) and HC (*n* = 11) revealed significantly *less* methylation of the *rDNA* promoter region in BPD compared with HC subjects, and hypomethylation of the *5*′*ETS* in BPD by trend. *PRIMA1* showed a *higher* methylation in BPD subjects (28).

Another GWA with PBC DNA was performed by Prados et al. (29) on BPD subjects affected with high levels of childhood adversity (*n* = 96) and subjects with MDD and a history of low levels of childhood adversity (*n* = 93). Uni- and multivariate analyses revealed significant methylation differences in a significant number of particular CpG sites associated with BPD compared with MDD subjects or related to childhood maltreatment, respectively. Contrasting BPD with MDD subjects, most significant results of multivariate analyses resulted in significantly different methylated CpG sites located e.g., within the gene coding for a ligand of Eph-related receptor tyrosine kinases (*EFBN1*), closely to the gene coding for a suppressor of cytokine signaling (SOCS) family member (*SPSB2*) and near the gene coding for a protein similar to mouse cystatin 9 (*CST9L*). Univariate analyses detected hypomethylated CpG sites in BPD i.e., near the encoding region of microRNA 124 (*miR124-3*), which targets several genes that have been described to be correlated with BPD (including *NR3C1*), near the gene coding for the WD repeat domain 60 (*WD60*), and a CpG site within the gene of the family with sequence similarity 163 member A (*FAM163A*). Many of the significantly hypermethylated sites were found on chromosome X. Targeting on CT, results of multivariate analyses with the CTQ revealed strong associations with loci within or near the genes of human homolog of the mouse p (pink-eyes dilution) (*OCA2*), microfibrillar-associated protein 2 (*MFAP2*), *CST9L*, E1A binding protein p400 (*EP400*), *KCNQ2*, alpha-2-macroglobulin-like 1 (*A2ML1*), 5′-nucleotide domain containing 2 (*NT5DC2*) and rabphilin 3A-like (*RPH3AL*). Univariate analyses yielded most significant alterations located within the gene coding for the interleukin 17 receptor A (*IL17RA*), in an intergenic region on chromosome 6p22.1, and closed by *miR124-3* and *miR137*. Methylation of *miR124-3* was associated with both severity of childhood adversity (higher methylation) and with BPD (lower methylation).

In summary, large part of the studies in PD focused on single genes, including *MAOA* in ASPD, and *BDNF, NR3C1, DRD2*, and *HTR3A* in BPD, or on a set of theory-driven suggestive genes (26). They revealed significant methylation differences in blood cell DNA of subjects with the respective PD. Within all independent gene-targeted studies, *NR3C1* hypermethylation was most frequently and consistently found to be associated with BPD; therefore, current results most strongly indicate *NR3C1* to be implicated in BPD. *NR3C1* further was the only gene that was as well affected in one of the GWA (29), however this was mediated by methylation differences of *miR124-3* which targets, among other genes, on *NR3C1*. In contrast, GWA studies, as performed by Teschler and Prados, found large number of genes that were differentially methylated in BPD, indicating a system-wide involvement in PD including genes associated with immune-response, cell-signaling or transcription control (27–29). The transcriptional relevance of the respective methylation differences was only verified by two authors. Thus, Checknita et al. found an indirect negative association of *MAOA* promoter hypermethylation with 5-HT serum level *in vitro* (20). Otherwise, Perroud et al. (21) didn't found an association of *BDNF* promoter methylation and the peripheral protein serum level of BDNF. All studies were based on DNA extracted from PBC, in most cases from whole blood cells, partly from only leucocytes (21–23), or selective from lymphocytes (19). However, the studies particularly differ in their design of study subjects and controls, and at least in the consideration of environmental factors that have impact on epigenetic modulation like early childhood adversity, which impedes a well-defined interpretation in most of the studies (Table 1, col. “Limitations”).

### Personality traits

Most original publications on methylation and personality traits were found with antisocial or aggressive features in connection with epigenetic alterations associated with the functionality of the serotonergic system or the hypothalamic–pituitary–adrenal (HPA-) axis functionality, which is the main neuroendocrine system for regulation of stress reaction and adaptation. Accordingly, theory-driven studies examined genes associated with these systems, such as the serotonin transporter gene (*SLC6A4*), dopamine and serotonin receptor genes (*DRD1, HTR1B, HTR1D, HTR3A*), *MAOA*, and *NR3C1* promoter exon 1F. With respect to its role in socio-affective perception and processing, some studies exist that pertain to antisocial behavior and oxytocin and oxytocin receptor genes (*OXT, OXTR*). Further, the role of cytokines and other factors were considered.

#### Antisocial traits

Consistent with a large body of literature that implicates the serotonergic system in the regulation of anxiety, aggression, and stress response, epigenetic alterations in the serotonin transporter gene (*SLC6A4*) were found to be related to a history of child sexual abuse as well as to antisocial behavior in adulthood by Beach et al. (30). The authors performed methylation analyses on the participant's peripheral blood lymphocyte DNA that was EBV transformed into lymphoblast cell lines. Participants (*n* = 155, all female) were gradually diagnosed by means of symptom score of ASPD. Child sexual abuse was highly associated with mean methylation level and methylation was highly significantly associated with symptoms of ASPD, thereby playing a modulating role to develop antisocial traits after childhood sexual abuse. Interestingly, influence of *SLC6A4* methylation on ASPD severity was impacted by genotype, since association of methylation with the ss and the sl genotype of *SLC6A4* was significant, but not with the ll genotype. These results suggest an aggravating effect of methylation in *SLC6A4* risk (s) alleles for ASPD, further, methylation was increasingly associated with ASPD in carriers with greater number of s alleles.

Moul et al. found antisocial traits being associated with genetic and epigenetic modulation of the rs11568817 SNP in the *HTR1B* promoter region (31). In an investigation with boys (*n* = 117) exhibiting callous-unemotional (CU) traits and antisocial behavior problems, and grouped by high and less strong CU traits, they found lower methylation levels of *HTTR1B* in saliva cell DNA in the high CU trait group. In turn, methylation was decisively moderated by rs11568817 SNP genotype and carried by two exclusive CpG sites (CpG12 and CpG14), which individual methylation levels were negatively associated with overall methylation levels in this gene region. The authors assume two ways of risk for high CU traits, first, carrier of the risk (minor) allele (s) with low levels of methylation at CpG sites 12 and 14 (what was associated with high overall promoter methylation) and second, carrier without risk allele but with high methylation at CpG 12 and CpG14 (what was associated with low overall methylation) and high expression of *HTR1B* (31).

In the same sample of participants stratified by CU score level and by age, the aforementioned research group analyzed methylation characteristics of the ocytocin receptor gene (*OXTR*) in PBC DNA. They revealed high CU traits being associated with increased methylation at two of the six analyzed CpG loci within the *OXTR* promoter and being correlated with decreased oxytocin blood levels. Yet, when divided in age group, these findings reached significance only in the older group (age 9–16 years), not in the prepubertal children (age 4–8 years) (here only by trend). Similarly, oxytocin serum levels significantly were negatively correlated with gene methylation level in the older boys (32).

Further, in a prospective, longitudinal study, Cecil et al. (18) studied 84 youth with early-onset and persistent conduct problems with regard to early life risks and to methylation changes of *OXTR* in PBC DNA. Clinical surveys took place at birth, age 7, 9, and 13 years, and collected an environmental risk score (kind and time of risk factor), diagnosis of conduct problems, CU traits and internalizing problems. Equally, methylation analyses were performed longitudinally with sampling at birth (cord blood), and at age 7 and 9 years (peripheral blood). Finally, for evaluation subjects were divided by severity of internalizing problems, collected by maternal reports at each study time.

Results for youth with *low* internalizing problems revealed that *OXTR* hypermethylation at birth was significant related to higher CU traits at age 13. *OXTR* methylation at birth further was associated with decreased experience of victimization during early childhood, specifically direct victimization. The only environmental risk factor associated with *OXTR* methylation at birth was a prenatal parental risk i.e., maternal psychopathology, criminal behaviors, and substance use. Within the youth with *high* internalizing problems, CU traits showed no significant association with *OXTR* gene methylation, but were positively correlated with prenatal, specifically interpersonal, risks like intimate partner violence or family conflicts. Interestingly, CU traits were negatively correlated with postnatal risks, specifically life events as the death of a relative, an accident or illness. These different correlation results are suggestive for discriminative pathways in the development of CU traits, the one epigenetically co-determined, and the other influenced by different environmental factors. If associated with *OXTR* hypermethylation at birth, CU traits may protect for internalizing problems and for experienced victimization, as discussed by the authors as a possible evocative correlation (18).

In view of ASPD symptoms, Philibert et al. (33) explored the *MAOA* VTNR polymorphism genotype and methylation status in EBV transformed lymphoblast cell lines with respect to lifetime symptom severity of ASPD and substance use disorder as defined as alcohol or nicotine dependence in a collective of 191 subjects (96 female). Methylation level was consistently higher in the female subjects than in males at each analyzed CpG site. There was only a statistical trend for women homozygous for the 3,3 allele showing higher average methylation than for the 4,4 allele. The research revealed no association between symptom counts for ASPD and methylation levels in neither men nor women (33).

With attention to multifaceted features in subjects with early-onset behavior problems as i.e., higher irritability, anxiety, impulsivity, possibly associated with an impaired hypothalamic-pituitary-adrenal (HPA) function, Dadds et al. (34) examined the methylation patterns of the *NR3C1* promoter exon 1F in whole blood and saliva cell DNA and the morning plasma cortisol levels within a study collective (*n* = 241, 51 female) with full criteria or features of conduct disorder (CD) or oppositional defiant disorder (ODD) in contrast to an Australian normative sample as control (35). Psychopathologic symptoms were scaled into more internalizing disorders in contrast to features of externalizing disorders like CU traits or conduct problems. Overall methylation levels of *NR3C1* exon 1 in blood and saliva DNA probes were associated with higher levels of internalizing disorders. Distinctly, CpG10 showed an association of increased methylation with higher scores of internalizing symptoms. In contrast, methylation levels of CpG3 and CpG4 in saliva DNA probes predicted externalizing severity (34). Peripheral morning cortisol plasma levels were associated with hypermethylation of CpG9 in PBC DNA only.

Additionally, Radtke et al. (36) investigated the mutual influences of childhood maltreatment history, methylation of the *NR3C1* promoter in peripheral blood lymphocytes and psychopathological load in subjects of a convenience sample (*n* = 46, 28 female). Their findings didn't indicate a significant correlation between average methylation of the *NR3C1* promoter region with childhood maltreatment severity or with scores of the collected psychometric data. Though, by single analyses they depicted two of 41 selected CpG sites that interacted with childhood maltreatment and vulnerability to psychopathology. One of the two mentioned CpG site was located in exon 1F promoter and its methylation was highly significantly correlated with the number of experienced childhood adversities according to the CTQ as well as with BPD symptoms. With performing a linear model the authors revealed an independently strong effect of both childhood maltreatment and methylation status of this CpG site on the development of BPD-associated symptoms, and they found an additive effect of both of these factors (36).

In summary, studies on epigenetic associations with antisocial traits all focused on single genes. Each one study revealed significant methylation modifications in the *SLC6A4* promoter (30) and in *HTR1B* (31), both in interaction with genotype variants of the concerned gene. Two studies focused on *OXTR*, with different results. In the first study, high CU traits were associated with *OXTR* hypermethylation, which was correlated with lower serum OXT level (32). In another, longitudinally performed study, *OXTR* hypermethylation at birth was associated with higher CU traits at prepubertal age in youth with low internalizing problems, but *OXTR* methylation was not associated with CU traits in youth with high internalizing problems, in which they were positively correlated with prenatal interpersonal risks and negatively with negative life events (18). This suggests a different (epi-)genetic pathogenesis of CU traits. In contrast, mean methylation of *MAOA* was not associated with ASPD symptom scores (33), nor was it significantly related to the *MAOA* VTNR genotypes. However, differentiated results exist in the two independent studies with *NR3C1*. Therefore, hypermethylation of distinct CpG-Sites within *NR3C1* promoter exon 1F, but not overall methylation, was associated with externalizing symptoms (34). This is consistent with the results of another study, in which *NR3C1* promoter exon 1F methylation showed no correlation with psychometric scores, but in consideration with childhood maltreatment both factors interacted in predicting vulnerability to psychopathology (36). Interestingly, *NR3C1* was most investigated in BPD (-> Borderline Personality Disorder). Only two studies considered ELA as an independent factor (30, 36). All studies fail to provide a healthy control group; most of the studies are restricted to only male or female study subjects. Only one study analyzed the transcriptional relevance of the detected methylation alterations by means of the protein level (32). Hence, the represented studies in antisocial and BPD-associated personality traits yet cannot provide a base for general conclusions.

#### Aggression

Within a 21 year longitudinal study on early onset aggression the research group Wang et al. specified children by severity and persistence of aggressive behavior during their age of 6–15 years and established four states of childhood aggression trajectories: the first consisted in subjects with no physical aggression at any time points (no physical aggression, NPA), a second in subjects with low to moderate aggression that declined between age 10–15 (low physical aggression, LPA), the third in subjects with high rates of aggression that subsequently declined (childhood-limited high physical aggression, CLHPA) and finally in subjects with consistently high physical aggression until age 15 (chronic high physical aggression, CPA).

Among the described study cohort, Wang et al. (37) studied *SLC6A4* methylation in comparison of healthy male young adult subjects with childhood-limited high physical aggression (CLHPA, *n* = 7) and those with no and low physical aggression in childhood, combining the latter as a control group (*n* = 18). They analyzed DNA extracted from isolated peripheral monocytes and in T cells. Not in T cells but in monocytes the authors found a significantly higher average methylation level across all CpG sites and the authors suggest monocytes to be more reliably in their association study. However, functional analysis in a luciferase assay showed significantly decreased *SLC6A4* gene transcriptional activity for both T cells and monocytes in the CLHPA group. Regarding particular CpG sites individually, methylation at CpG11 and CpG12 were highly positively correlated and significantly higher in the CLHPA group. Individual methylation at CpG5 and CpG6 were highly correlated and higher in the CLHPA subjects. No association was found between mean methylation levels of *SLC6A4* with mRNA level, and with the existing serotonin-transporter-linked polymorphic region (5-HTTLPR) genotype. Amendatory, in a cranial PET scan, the authors observed a significant negative association between methylation levels at CpG 11 and CpG12 in T cells or at CpG5 and CpG6 in monocytes, respectively, and *in vivo* 5-HT synthesis in the lateral left and right orbitofrontal cortex (OBFC), indicating long-term functional effects *in vivo*. The authors discuss their results with caution due to the small sample size (37). In connection with the aforementioned study, Provençal et al. (38) compared male adult subjects with chronic high physical aggression (CPA, *n* = 8) with subjects of a conjoint cohort of the other three above defined aggression groups (37) which had developed normal aggression trajectories in adulthood (*n* = 12). They surveyed the methylation status in extracted DNA from peripheral monocytes and T cells with a comprehensive microarray encompassing the entire genomic region including the cytokines IL1A, IL6, IL8, IL4, and IL10 and the transcription factors nuclear factor kappa B subunit 1 (*NFkB1*), nuclear factor of activated T cells 5 (*NFAT5*), signal transducer and activator of transcription 6 (*STAT6*) in both study groups. The results showed significant methylation differences concerning each of the analyzed cytokine gene loci. In CPA subjects overall methylation levels were significantly decreased in *IL1A, IL4, IL6*, and *IL8*, and were significantly increased in *IL10*. Methylation was positively correlated with serum protein levels of IL1A (significant), IL4, and IL-6, and was negatively correlated for IL-8 and IL-10, respectively. In regard to the transcription factors, two regions closely located to the TSS of each *STAT6* isoform were significantly higher methylated in the CPA group, which was significantly associated with lower transcription of IL4. Next, Provençal et al. conducted a GWA (39) in connection with CPA by comparing male young adult subjects with early development of CPA (*n* = 8) with subjects without any history of high physical aggression in childhood (NPA, *n* = 16) according to the former study definition (37, 38). They analyzed DNA from peripheral T cells and found early onset CPA being associated with clustered and genome-wide methylation differences at 900 sites within 448 distinct gene promoters. The detected genes comprised several that have been associated with aggression earlier: arginine vasopressin receptor 1A (*AVPR1A*), serotonin receptor 1D (*HTR1D*) and glutamate metabotropic receptor 5 (*GRM5*) genes with less methylation in the CPA group, and the dopamine receptor D1 gene (*DRD1*) and *SLC6A3* with higher methylation in the CPA group. In total, most functional categories of genes with different methylation in CPA included behavior (hyperactivity), metabolic and neurological diseases, inflammatory response (chemotaxis and phagocytes), cellular growth and proliferation, and gene expression (transcription factors, signal transducer as *STAT6*). Concerned specific canonical pathways included cytokine signaling and G-protein coupled receptor signaling.

In a further GWA Guillemin et al. studied methylation aberrancies in DNA from isolated peripheral T-cells in female subjects with CPA trajectories since childhood (*n* = 5) compared to women without any aggression history (*n* = 16) (40). A total of 917 probes corresponding to 430 distinct gene promoters were differentially methylated, of which many are involved in immune and inflammatory responses such as *FOS, GATA1, GATA3*, hepatocyte growth factor (*HGF*), *TNF, IFNG, IL1A, IL1B, IL10, IL13, IL17R*, and *IL18*. Thereof, some had previously been shown to be associated with aggressive behavior, including Tryptophan hydroxylase 2 (*TPH2*), *NR3C1*, and Corticotrophin-releasing hormone-binding protein (*CRHBP*), all of them were significantly less methylated in the CPA subjects.

In a second step, the researchers compared their results with their previous data of a GWA in male subjects out of the same underlying longitudinal study by Provençal et al. (39). Methylation levels of 31 gene promoters emerged to associate with physical aggression in both women and men and therewith constituted a significant overlap, including *TPH2, CRHBP*, and *NR3C1*. In the region within the zinc finger protein 366 gene (*ZNF366*) promoter, aggression-associated methylation was differentially directed in both women and men (40).

Van Dongen et al. (41) studied methylation differences with respect to aggressive behavior in 40 monozygotic twins (20 pairs) highly discordant for aggressive traits. They performed a GWA with PBC DNA and received about 24 methylation sites with high methylation difference, yet differences were generally twin pair-specific; thus, statistically no genome-wide significant methylation differences were identified in this sample.

Subsuming the main results of studies on aggressive traits, four of the five studies derived from the same study group. Two studies were theory-driven and gene-targeted. Their results indicate increased methylation of *SLC6A4* in healthy men with a history of CLHPA with implications for the transcriptional activity *in vitro*, and showed associations with brain 5-HT in the left lateral OBFC (37). A more comprehensive genome region analysis including *IL1A, IL4, IL6, IL8, IL10*, and transcription factors *NFkB1, NFAT5*, and *STAT6* revealed significant methylation differences in men with CPA in transcriptional relevant sites near or within each of the investigated genes. However, correlations with interleukin serum plasma levels only were significant for *IL1A*. Many of the identified methylation aberrances concerned sites outlying the intrinsic gene sequences, underlining the relevancy of methylation analyses that are not restricted to targeted gene sequences (38). A complementary study in women with CPA revealed similar results for immune and inflammatory genes (40). GWA results confirmed genome-wide involvement of methylation differences with respect to aggressive traits in men (39). The mentioned studies were performed in a small sample size of 8 (men) and 5 (women) subjects, respectively, in the aggression group. None of the studies considered the experience of childhood trauma.

Overall, the depicted studies confirm the need of genome-wide methylation assays, or at least a focus on a comprehensive set of functional associated genes. Future studies should consider childhood trauma, and specify an applicable control group.

#### Impulsiveness

By performing a GWA Ruggeri et al. (42) studied methylation aberrancies in PBC DNA of monozygotic twin pairs (*n* = 18) discordant for alcohol use disorder. The authors received 77 differentially methylated regions associated with 62 genes. Replication of these findings in a microarray only revealed significant methylation changings at one CpG site in the 3′UTR of 3′-protein-phosphatase-1G gene (*PPM1G*). Hypermethylation in *PPM1G* was positively correlated with lower mRNA levels of *PPM1G* and significantly associated with early escalation of alcohol use, as well as with increased impulsiveness. Further analyses evidenced that *PPM1G* hypermethylation was independently associated with both trait impulsiveness and right subthalamic nucleus activation, presumptively due to an increased effort to carry out control inhibition. Since *PPM1G* is assembled with five SNP's, the authors performed regression analyses which could rule out significant influences of genotype variations on *PPM1G* methylation. Further, no correlation was found for the SNP's with the trait impulsiveness (42).

Studies on personality traits are summarized in Table 2.

**Table 2:** Studies on epigenetics in personality traits.

**Author**	**Year**	**Trait**	**Gene**	**Co-factors**	**Results**	**Sample**	**Tissue/DNA**	**Limitations**
Beach et al. (30)	2011	Antisocial	*SLC6A4*	5-HTLPR genotype, SA	No significant association of child SA on ASPD symptom score (only by trend). Significant association of *SLC6A4* methylation level with child SA and with ASPD symptom score, resp. Impact of genotype: significant correlation between *SLC6A4* promoter methylation and ASPD in subjects with ss genotype, and with sl genotype, but not with ll genotype. Methylation potentiated the effect of *SLC6A4* risk alleles.	*Subjects:* adult female participants of the IAS (*n =* 155). *Diagnostics:* Retrospective clinical interview on childhood sexual abuse, ASPD symptoms: SSAGA-II (consistent with DSM-IV). *Methods:* DNA bisulfite treatment, selected methylation analysis of 71 CpG sites within the *SLC6A4* promoter by quantitative mass spectroscopy DNA sequencing for genotyping.	EBV-transformed lymphoblast cell lines	Only female subjects. No HC.
Moul et al. (31)	2015	Antisocial	*HTR1B*	SNP genotype	*Genotype and CU traits:* high CU subjects were significantly more likely to be heterozygous for the minor (risk) allele. *Genotype and methylation:* genotype was significantly associated with overall methylation level of *HTR1B*. Significantly lower methylation levels in minor homozygotes compared to heterozygotes or major homozygotes. *Methylation and CU traits:* No main effect of genotype or *HTR1B* methylation on CU traits, but genotype by methylation was a significant predictor of CU traits. In heterozygotes CU traits were significantly positively correlated with *HTR1B* methylation. In major homozygotes CU traits were significantly negatively correlated with *HTR1B* methylation. Individual CpG sites: 2 of 19 CpG sites (CpG 12 and 14) interacted with the genotype as significant predictors for CU traits, and correlated negatively with overall *HTR1B* methylation. CpG12: significant negative correlation between methylation and CU traits in heterozygotes, significant positive correlation in major homozygotes.	*Subjects:* male children (*n =* 117) with CU traits, aged 3–16 years. Subjects were grouped by CU traits (see text). Comorbidity: ODD/CD *n =* 57), ADHD (*n =* 33), anxiety/depression (*n =* 11), ASD (*n =* 15). *Diagnostic:* DSM-IV, DISCAP, APSD, SDQ, QFE. *Methods:* DNA sequencing of *HTR1B* SNP rs11568817genotype by PCR and mass spectrometry analysis. Selected methylation quantification by quantitative mass spectroscopy of three CpG rich regions surrounding the SNP (19 CpG).	Saliva	Only male subjects. No HC. No consideration of ELA.
Dadds et al. (32)	2014	Antisocial	*OXTR*		In the whole sample, higher CU traits were associated with higher methylation levels at two particular CpG sites of *OXTR* and with lower OXT blood levels. Methylation level and OXT blood level were inversely correlated. If grouped by CU severity and by age, in the older group methylation was significantly higher and OXT serum levels significant lower in the high-CU subjects. In the prepubertal group methylation and OXT blood levels varied similar but only by trend.	*Subjects:* male children (*n =* 156), aged 4–16 years with conduct problems (full or symptoms of ODD or CD according to DSM-IV) with a severity >3 (scale 0–6) in to the DISCAP. Prepubertal group (age 4–8 years), pubertal group (age 9–16 years). Subjects were grouped by CU traits (see text). *Diagnostics:* DSM-IV, DISCAP, APSD (level of CU), SDQ, QFE. *Methods:* genotyping by PCR and mass spectrometry analysis. Selected methylation analysis of 11 CpG sites within an *OXTR* CpG island (Chr3: 8810680- 8810890) by quantitative mass spectroscopy (subsample *n =* 98). OXT plasma levels (subsample *n =* 37), OXT extraction by cold acetone ether, quantification by radioimmunoassay.	PBC	Only male subjects. No HC. No consideration of ELA.
Cecil et al. (18)	2014	Antisocial	*OXTR*		*Youth with low internalizing problems:* OXTR methylation at birth was related to higher CU traits at age 13 and to decreased experience of direct victimization during early childhood. OXTR methylation at birth was positively associated with prenatal parental risks (maternal psychopathology, criminal behaviors, substance use) and was temporal more stable than in youth with high internalizing problems. *Youth with high internalizing problems:* CU traits showed no association with OXTR methylation, but were positively correlated with prenatal, especially interpersonal risks (intimate partner violence, family conflict). CU traits were negatively associated with postnatal risks, specifically life events (death of relative, accidents, illness).	*Subjects:* youth (*n* = 84), aged 0–13 years with early-onset and persistent conduct problems, divided in groups with low (*n =* 39) and high level (*n =* 45) of internalizing problems. *Diagnostics at birth, age 7, age 9, age 13:* Strengths and Difficulties Questionnaire (subscale “conduct problem”), Environmental risk score (by maternal reports), Development and Well-being Assessment (internalizing problems, by maternal reports), CU traits questionnaire oriented on Antisocial Process Screening Device (by mothers report). *Methods:* cord blood (at birth) and peripheral blood (at age 7+9). DNA bisulfite treatment, BeadChip technology for methylation quantification. Selected methylation analysis of 12 CpG sites within an *OXTR* CpG island (hg19; chr3:8808962–8811280).	PBC	No HC. No consideration of ELA.
Philibert et al. (33)	2008	Antisocial	*MAOA*	VTNR genotype	No significant association of overall methylation of *MAOA* and symptom counts for ASPD in women or men. Each particular CpG site showed higher methylation in female subjects. By statistical trend women homozygous for the 3,3 allele have higher average methylation of *MAOA* than 4,4 allele subjects.	*Subjects:* adult participants of the IAS (s.a.) (*n =* 191, 96 female). *Diagnostics:* DSM-IV. *Methods:* DNA bisulfite treatment, PCR amplification and selected methylation analysis of two CpG-rich regions (10 and 70 CpG-sites) of *MAOA* by quantitative mass spectroscopy. DNA Sequencing of *MAOA* VTNR genotype.	EBV-transformed lymphoblast cell lines	No consideration of ELA.
Dadds et al. (34)	2015	Antisocial	*NR3C1*		*NR3C1* overall methylation level (blood and saliva) was associated with higher levels of internalizing disorders. Hypermethylation (blood and saliva) of CpG10 was associated with higher internalizing symptoms. CpG3 and CpG4 hypermethylation (saliva) predicted externalizing severity. Elevated morning cortisol plasma levels were associated with increased methylation of CpG9 (blood).	*Subjects:* children (*n =* 241, 51 female), aged 4–16 years with conduct problems (full or symptoms of ODD or CD according DSM-IV), referred to the Australian Normative Sample as control (19). *Diagnostics:* DSM-IV, SDQ, DISCAP. Comorbidity: ODD/CD 66%, ADHD 26%, anxiety/depression 6%, ASD 5%. *Methods:* Genotyping and selective methylation analysis of *NR3C1* promoter exon 1F (12 CpG sites) by mass spectroscopy. Morning cortisol plasma levels were quantified by chemiluminescent enzyme immunoassay.	PBC (*n =* 90), saliva (*n =* 151)	HC only for diagnostic data. No consideration of ELA.
Radtke et al. (36)	2015	Antisocial	*NR3C1*	CM	CM and *NR3C1* promoter methylation was strongly correlated with an increased vulnerability to psychopathology. No significant correlation of *NR3C1* methylation neither with CM nor with applied psychometric measurements. Individual CpG sites: methylation at CpG cg17860381 (located in exon 1F) was significantly associated with the number of experienced CM and with severity of BPD symptoms (nearly significantly with depression). It was positively (not significant) correlated with ODD-associated symptoms and behavioral strength and difficulties. Interaction of CM and *NR3C1* methylation: CM and *NR3C1* mean methylation significantly interacted in predicting psychological health. CM and methylation of CpG cg17860381 showed an additive, but no interactive impact on the development of BPD-associated symptoms.	*Subjects:* convenience sample from the local community (*n =* 46, 28 female), aged 11–21 years. *Diagnostics:* KERF-I (specifies CM in 8 dimensions: parental PA, parental EA, SA, witnessed physical violence toward parents/siblings, peer physical violence, PN and EN), BPD: BSC-23, HSC-25 (depression, anxiety), MINI (ODD/OD). KIDSCREEN-53, SDQ. *Methods:* DNA bisulfite treatment, GWA by Human Methylation 450K Array, covering 41 CpG sites within the *NR3C1* promoter.	Lymphocytes (isolated by Ficoll gradient)	No group design.
Wang et al. (37)	2012	Aggression	*SLC6A4*	5-HTLPR genotype	*T-cells:* no significant differences in *SLC64A* average methylation level of CLHPA subjects and controls, but high variability in methylation status at individual CpG sites. Methylation at CpG11 and CpG12 was significantly higher in CLHPA subjects. *Monocytes:* average methylation level was higher in CLHPA. In particular methylation at CpG5 and CpG6 was significantly increased in CLHPA subjects. No association of methylation levels and 5-HTTLPR genotype. *Functional analysis:* Hypermethylation of SLC6A4 significantly decreased gene transcriptional activity in the luciferase assay. *5-HT synthesis:* methylation of CpG11 and CpG12 in T-cells, and of CpG5 and CpG6 in monocytes, respectively, was significantly negatively correlated with 5-HT synthesis in the lateral left OBFC.	*Subjects:* healthy male young adults with none or low aggression (LPA) in childhood (controls, *n =* 18) and CLHPA (*n =* 7). Tissue: T-cells, monocytes. *Diagnostics:* based on longitudinal observation data collected annually between age 6 and 15, clinical diagnostic, SDQ. *Methods:* DNA bisulfite treatment, PCR amplification, quantitative pyrosequencing of *SLC6A4* promoter (24 CpG sites). Genotyping of *5-HTLPR*. *In vitro* functional analysis (luciferase assay). PET: tracer alpha-[(11)C]methyl-L-tryptophan ((11)C-AMT) for 5-HT synthesis analysis. Voxel of interest: hippocampus, lateral and medial OBFC, ncl. caudate.	Monocytes, CD3+ T cells (density centrifugation)	Only clinically healthy subjects, only men. No consideration of ELA.
Provençal et al. (38)	2013	Aggression	*IL1A, IL4, IL6, IL8, IL10, NFkB1, NFAT5, STAT6*		Methylation status of the 5 cytokine and 3 transcription factor gene loci was different in each analyzed CpG site in either T-cells (20 loci) or monocytes (28). In CPA subjects mean methylation levels were significantly decreased in *IL1A, IL4, IL6*, and *IL8*, and increased in *IL10*. Methylation levels were positively correlated with Plasma protein level for IL1a, IL-4, IL-6, and negatively correlated for IL-8 and IL10. Two regions closed to the TSS of each *STAT6* isoform were significantly hypermethylated in the CPA group. Many of the revealed methylation deviant loci were associated with DNAse clusters.	*Subjects:* male young adults with CPA (*n =* 8), controls (*n =* 12) recruited from all the trajectory groups NPA, LPA, CLHPA. *Diagnostics* and definitions as described earlier (22). *Methods:* Methylated DNA immunoprecipitation with microarray hybridization for *IL1A, IL4, IL6, IL8, IL10, NFkB1, NFAT5, STAT6*. Microarray validation: DNA bisulfite treatment, PCR amplification, quantitative pyrosequencing (48 CpG sites).	Monocytes, CD3+ T cells (density centrifugation)	Only men. No consideration of ELA.
Provençal et al. (39)	2014	Aggression	genome-wide		High physical aggression was associated with clustered and genome-wide variations in promoter methylation. Within the plenty, 227 gene loci were significantly more methylated in the control group (including 10 micro RNA promoters) and 171 were significantly more methylated in the CPA group (including 2 microRNA promoters). Five of the genes were already described to be linked with aggression: *AVPR1A, HTR1D, GRM5* were less methylated in the CPA group and *DRD1* and *SLC6A3* were hypermethylated.	*Subjects:* male young adults with CPA (*n =* 8), controls with no (never) history of high physical aggression from age 6–15 (*n =* 12). *Diagnostics* and definitions as described earlier (22). *Methods:* GWA: Methylated DNA immunoprecipitation with microarray hybridization. Microarray validation: by quantitative real-time PCR on immunoprecipitated DNA and by pyrosequencing of bisulfite treated DNA.	Monocytes, CD3+ T cells (density centrifugation)	Only men. No consideration of ELA.
Guilleminet al. (40)	2014	Aggression	genome-wide		Significant association of CPA with methylation aberrances in a plenty of genes, many involved in immune and inflammatory responses such as *FOS, GATA1, GATA3, HGF, TNF, IFNG, IL1A, IL1B, IL17R, IL10, IL13*, and *IL18*. Methylation differences were aggression-associated in 31 promoter sites in both woman and men, i.a. *TPH2, CRHBP, NR3C1* (less methylated in the CPA group). One CpG site was significantly associated with aggression in both women and men, but was differentially deviant methylated (*ZNF366* promoter region).	*Subjects:* female young adults with CPA (*n =* 5), controls without any aggression history (*n =* 16). *Diagnostics* as described earlier (22). *Methods:* GWA: Methylated DNA immunoprecipitation with microarray hybridization. Microarray validation: gene-specific by quantitative real-time PCR on immunoprecipitated DNA and genome-wide by using BeadChip technology. Retrospective comparison with results in male young adults (28)	CD3+ T cells (density centrifugation)	Only women. No consideration of ELA.
van Dongen et al. (41)	2015	Aggression	genome-wide		Large number of differently methylated loci, none resisting Bonferroni correction. In the subsample of 20 MZ twin pairs discordant for aggressive traits, methylation differed at 24 sites which were twin pair-specific and without statistically relevance in the whole assay. Suggestive CpG sites were i.e. gene loci near *RAB39, SIGLEC10, PREP*.	*Subjects:* subjects from the Netherlands Twin Register. First GWA: 2029 subjects (1247 MZ, 663 DZ, parents and other siblings). Second GWA: 20 MZ pairs, highly discordant for aggression. *Diagnostics:* ASR. *Methods:* DNA bisulfite treatment, GWA BeadChip technology^*^.	PBC	No consideration of ELA.
Ruggeri et al. (42)	2015	Impulsiveness	genome-wide, *PPM1G*		GWA resulted in 77 differently methylated sites. In the replication array, only methylation of *PPM1G* was significantly different and was positively associated with increased impulsiveness. PPM1G hypermethylation was significantly associated with lower mRNA levels. *PPM1G* hypermethylation was independently associated with both trait impulsiveness and right subthalamic nucleus activation.	*Subjects:* MZ twin pairs (*n =* 18), aged 24y. *Diagnostics:* DSM-III-R, RAPI, SURPS, PDS. *Methods:* GWA. Validation assay: bisulfite PCR for genes *PPMG1, INS*-*IGF2, FMN1, SEPHS2, SLC6A3, AIM1, OPRL1*, and *PIPOX*. Functional analysis: mRNA quantification by hybridizing labeled cRNA on BeadChip technology. fMRI: BOLD brain analysis during an inhibitory control task	PBC	No consideration of ELA.

5-HT, 5-hydroxytryptophane (serotonin); 5-HTTLPR, serotonin-transporter-linked polymorphic region; ADHD, attention deficit hyperactivity disorder; APSD, Antisocial Process Screening Device; ASD, Autism spectrum disorder; ASPD, Antisocial personality disorder; ASR, ASEBA Adult Self Report; AVPR1A, arginine vasopressin receptor 1A gene; BOLD, blood-oxygen-level-dependent response; BPD, borderline personality disorder; BSC-23, Borderline Symptoms Checklist-23; CD, conduct disorder; CLHPA, childhood-limited high physical aggression; CM, childhood maltreatment; CPA, chronic high physical aggression; CRHBP, CREB binding protein gene; CU, callous-unemotional; DISCAP, Diagnostic Interview Schedule for Children, Adolescents and Parents; DRD1, dopamine receptor D1 gene; DZ, dizygotic; EA, emotional abuse; ELA, early life adversity; EN, emotional neglect; FOS, fos proto-oncogene AP-1, transcription factor subunit; GATA1/GATA3, GATA binding protein 1/3; GRM5, glutamate metabotropic receptor 5 gene; GWA, genome-wide methylation analysis; HC, healthy control; HGF, hepatocyte growth factor gene; HSC-25, Hopkins Symptoms Checklint-25; HTR1B/HTR1D, serotonin receptor 1B/1D gene; HTR3A, serotonin receptor 3A gene; IAS, Iowa Adoptee Study; IFNG, interferon gamma gene; IL1A, (1B/4/6/8/10/13/17A/18) interleukin 1A (1B/4/6/8/10/13/17A/18) gene; IL17RA, interleukin 17 receptor A gene; IPV, interpersonal violence; KERF-I, German version of MACE (Maltreatment and Abuse Chronology of Exposure); KIDSCREEN-53, children questionnaire of health-related quality of life; LPA, low physical aggression (subjects); MAOA, monoamine oxidase A gene; MINI, Mini-International Neuropsychiatric Interview; MZ, monozygotic; NR3C1, glucocorticoid receptor gene; OBFC, orbital frontal cortex; ODD, oppositional-defiant disorder; OXT, oxytocin; OXTR, oxytocin receptor gene; PA, physical abuse; PBC, peripheral whole blood cells; PDS, Pubertal Development Scale; PN, physical neglect; PPM1G, 3'-protein-phosphatase-1G gene; PREP, prolyl endopeptidase gene; PTSD, posttraumatic stress disorder; QFE, Quality of the Family Environment questionnaire; RAB39, RAS oncogene family 39 gene; RAPI, Rudgers Alcohol Problem Index; SA, sexual abuse; SDQ, Strengths and Difficulties Questionnaire; SIGLEC10, Sialic Acid Binding Ig Like Lectin 10 gene; SLC6A3, dopamine transporter gene; SLC6A4, serotonin transporter gene; SNP, single nucleotide polymorphism; SSAGA-II, Semi-Structured Assessment for the Genetics of Alcoholism; STAT6, signal transducer and activator of transcription 6; SURPS, Substance Use Risk Profile Scale; TPH2, tryptophan hydroxylase 2 gene; VTNR, variable number tandem repeats; ZNF366, zinc finger protein 366. ^*^Results were corrected for white cell counts.

## Discussion

The objective of this review was to summarize the first complete number of current original publications on human personality disorders and personality traits in connection with epigenetic modifications and to discuss the results as to pathogenetic impact and treatment-relevant insights. Remarkably, literature search yielded no single study of epigenetic evaluations in narcissistic, histrionic, anankastic, avoidant, dependent, eccentric, paranoid, schizoid and schizotypal personality aspects. Neither there was any result with any keyword on histone modification, nor results with studies on microRNA, but only on methylation aberrancies. Aggression and antisocial traits were prevalently explored, possibly preferred since these behavioral characteristics have a high genetic loading [e.g., (43)].

Most of the theory-driven studies focussed on gene loci involved in the serotonergic, dopaminergic or noradrenergic neurotransmitter system, e.g., serotonin receptor and transporter, dopamine receptor, and MAOA. Further crucial interest was in epigenetic modulation of the functionality of neurotrophic factors, the HPA-axis circuit, and the oxytocinergic system. This seems consequent, since these gene products are constitutive for brain function. Thusly, MAOA, a X-chromosomal encoded enzyme, is responsible for the oxidative breakdown of monoamine neurotransmitters, that is in the brain specifically serotonin, epinephrine, norepinephrine, and dopamine. Mutations in the MAOA gene with impaired gene activity lead to excess of serotonin and norepinephrine which is associated with disturbed control of an affected subject's impulsivity and aggression (NIH, Genetics Home Reference). BDNF is involved in growth, maturation and maintenance of nerve cells and plays a crucial role in building up synapses and in synaptic plasticity. Polymorphisms in the BDNF gene are associated with an increased risk of psychiatric disorders like bipolar disorder, anxiety, and eating disorders. However, results from genome-wide association studies refer to epigenetic alterations of genes that, besides neurofunctional genes, include genomic regions affecting genes involved in inflammation, cell-signaling, metabolism, and genes coding for proteins of the transcriptional machinery themselves (27, 29, 39–42). Methylation differences therefore represent a complex pattern that precludes feasible functional verification tests of the concerned gene loci, as it complicates a conclusive interpretation. The here reviewed GWA studies all, except for Prados et al., lack the consideration of additional factors possibly influencing the epigenetic configuration, especially early childhood experiences, which is also of main interest in understanding the development of a PD. For example, early life adversity was associated with methylation of *SLC6A4* that in turn showed association to brain structure and function (44). In contrast, most of the studies on PD and single gene analyses respected childhood trauma as a confounding factor in their epigenetic analyses. According to previous data, these gene targeted studies confirmed the relevance of the serotonergic system for affective regulation and revealed methylation aberrances of the serotonin transporter (*SLC6A4*) and receptor genes (*5HTR1B, 5HTR2A*, and *5HTR3A*) to be linked with antisocial traits (30, 31) and BPD (25, 26). As well for *DRD2*, methylation status was shown to be associated with BPD (24). Based on its effect on the pathway of each serotonin, dopamine and norepinephrine, epigenetic changes of *MAOA* congruently were found to be associated with antisocial personality traits, ASPD and BPD (19, 20, 26, 33). Studies also indicate a significant role of *NR3C1* promoter exon 1 methylation status in antisocial traits, as well as, in combination with early life adversity, in BPD (22, 26). Indeed, with the insight in a genome-wide involvement of epigenetic modified gene loci, the validity of the studies on particular genes today seems to be restricted.

Aside from a general association of epigenetic changes in personality disorders and traits, the study results highly differ regarding the particular gene sites of epigenetic differences. One major reason could be the lack or the incomparability of the respective control group in each study. Another methodical deficit is the inconstant consideration of environmental factors, particularly early child adversity. Epigenetically vulnerable influences further include exposure to intimate partner violence during pregnancy as well as mood, smoking or diet habits of the mother during pregnancy, and stress of the subject as a fetus or in its early life (45–49). Notably, in many studies, the sample size is very small and might impact the results.

All studies used extracted DNA from peripheral blood cells for methylation analyses, albeit different cell fractions, preferably whole blood cells, leucocytes, lymphocytes, T cells. There is evidence that peripheral mononuclear cells, particularly lymphocytes, show similar epigenetic pattern with brain tissue [i.e., (9)], and the use of peripheral blood cells has gained acceptance for epigenetic studies of neuropsychiatric disorders. But results also indicate differences of epigenetic patterns between blood leucocyte lines (37, 38) which also could account for discrepancies within the studies. Future studies should consider cell type and preparation from whole blood, since cell stress can cause epigenetic changes in the cells (50).

High requirements in the technical methods and in an expedient study design make the disentangling of personality-specific methylation patterns a sensitive challenge, especially since there might be more than one way to (epi-)genetically forward the development of a PD. This also might be a reason for the diversity of the final clinical epiphenotypes of these disorders. Conversely, the variety of pathogenesis and of the individually developed clinical shapes impedes the attribution of possibly identified genetic or epigenetic aberrances to a specific personality trait or disorder.

If the functional attribution of epigenetic aberrations to personality disorders is so highly impeded, which benefit can we gain in doing so?

The possibility of epigenetic subtyping helps to assess the individual pathophysiologic condition as well as to improve deductive therapeutic approaches. The identification of someone's epigenetic risk profile could initiate efforts to establish standards for intensified clinical watching in afflicted children, and methods for early parental coaching or for premature intervention in order to prevent further development of a full-blown clinical picture of a PD. As impressively shown by Cecil et al., divergent epigenetic conditions within psychopathologically similar subjects can elicit different implications for the individual treatment requirements (18).

As in other mental disorders, epigenetic patterns can help to predict medical response. Thus, in depression, selected methylation aberrations could predict different therapy response to escitalopram and between escitalopram and nortriptyline (51, 52). In BPD, methylation levels of *APBA3* and *MCF2* were predictive for psychotherapy outcome (53).

In cancer, advanced therapy today comprises epigenetic drugs targeting i.e., on silenced tumor suppressing genes. Due to the plurality and individual diversity of involved genes in psychiatric disorders, the use of demethylating drugs cannot be targeted to distinct genes. Adverse effects with general hypomethylation might conflict with the aimed benefits. But there is evidence that methyldonor components as valproic or folic acid have positive therapeutic effects in mental disorders by themselves as well as by increasing the effect of fluoxetine (54–57).

Aside from their primary neurobiological effect, psychotropic drugs can further exert direct epigenetic effects. Studies on antidepressants and antipsychotic drugs i.e., evidenced their possibility to modulate the epigenome by acetylation of gene-associated histones, by methylation changes in dopamine pathways (58), by increasing expression of DNA-methyltransferases, and by inducing chromatin remodeling (58, 59). Methylation aberrations also can be remodulated by psychopharmacological treatment or psychotherapy (60, 61).

These merely exemplary findings suggest a high dynamic in epigenetic processes in mental disorders and their course. The interaction of psychotropic drugs and other therapeutic interventions with epigenetic remodeling seems still understudied. Finally, the fact of this interaction then could be utilized as an intraindividual control of lasting therapy response.

## Implications and future options

The pathophysiological principle of gene-environment-interaction not only explains the obvious differences in the severity or combination of a person's personality traits but further implicates the existence of genetic and epigenetic risk and protective factors during the formation of the ultimate personality structure. Hitherto epigenetic studies underline the impact of early life adversity on the multifactorial pathway to PD. They reveal several relevant gene loci that are epigenetically affected in PD but differ in between the studies. This can partially be explained by the multifactorial and multi-step genesis of each PD, leading to different pathogenetic subtypes.

Epigenetic analyses in connection with PD represent a complex, but suitable amendment in the elucidation of personality development, and pose as valuable diagnostic step in the specification of an individual's premorbid risks, and for the development of individually tailored therapeutic strategies. They might play a valuable role in the future design and observation of early and personalized therapeutic intervention and thus could help to prevent the unfolding in severe dysfunctional conduct or personality disorder in risk subjects.

## Author contributions

DG conducted the full literature search process, read all found articles, wrote the manuscript. KK critically overworked focus and manuscript. TH and HF provided expert advice in epigenetics. JK and TF reviewed the manuscript and collaborated in the interpretation of the results and for the discussion.

### Conflict of interest statement

The authors declare that the research was conducted in the absence of any commercial or financial relationships that could be construed as a potential conflict of interest.
